# Considerations for an *In Vitro*, Cell-Based Testing Platform for Detection of Drug-Induced Inotropic Effects in Early Drug Development. Part 2: Designing and Fabricating Microsystems for Assaying Cardiac Contractility With Physiological Relevance Using Human iPSC-Cardiomyocytes

**DOI:** 10.3389/fphar.2019.00934

**Published:** 2019-08-29

**Authors:** Alexandre J. S. Ribeiro, Brian D. Guth, Michael Engwall, Sandy Eldridge, C. Michael Foley, Liang Guo, Gary Gintant, John Koerner, Stanley T. Parish, Jennifer B. Pierson, Mathew Brock, Khuram W. Chaudhary, Yasunari Kanda, Brian Berridge

**Affiliations:** ^1^Division of Applied Regulatory Science, Office of Clinical Pharmacology, Office of Translation Sciences, Center for Drug Evaluation and Research, US Food and Drug Administration, Silver Spring, MD, United States; ^2^Department of Drug Discovery Sciences, Boehringer Ingelheim Pharma GmbH & Co KG, Biberach an der Riss, Germany; ^3^PreClinical Drug Development Platform (PCDDP), North-West University, Potchefstroom, South Africa; ^4^Safety Pharmacology and Animal Research Center, Amgen Research, Thousand Oaks, CA, United States; ^5^Division of Cancer Treatment and Diagnosis, National Cancer Institute, National Institutes of Health, Bethesda, MD, United States; ^6^Department of Integrative Pharmacology, Integrated Sciences and Technology, AbbVie, North Chicago, IL, United States; ^7^Laboratory of Investigative Toxicology, Frederick National Laboratory for Cancer Research, Frederick, MD, United States; ^8^Health and Environmental Sciences Institute, Washington, DC, United States; ^9^Department of Safety Assessment, Genentech, South San Francisco, CA, United States; ^10^Global Safety Pharmacology, GlaxoSmithKline plc, Collegeville, PA, United States; ^11^Division of Pharmacology, National Institute of Health Sciences, Kanagawa, Japan; ^12^National Toxicology Program, National Institute of Environmental Health Sciences, Research Triangle Park, NC, United States

**Keywords:** microenvironment, cellular alignment, sarcomere, co-culture, electrical stimulation

## Abstract

Contractility of the myocardium engines the pumping function of the heart and is enabled by the collective contractile activity of its muscle cells: cardiomyocytes. The effects of drugs on the contractility of human cardiomyocytes *in vitro* can provide mechanistic insight that can support the prediction of clinical cardiac drug effects early in drug development. Cardiomyocytes differentiated from human-induced pluripotent stem cells have high potential for overcoming the current limitations of contractility assays because they attach easily to extracellular materials and last long in culture, while having human- and patient-specific properties. Under these conditions, contractility measurements can be non-destructive and minimally invasive, which allow assaying sub-chronic effects of drugs. For this purpose, the function of cardiomyocytes *in vitro* must reflect physiological settings, which is not observed in cultured cardiomyocytes derived from induced pluripotent stem cells because of the fetal-like properties of their contractile machinery. Primary cardiomyocytes or tissues of human origin fully represent physiological cellular properties, but are not easily available, do not last long in culture, and do not attach easily to force sensors or mechanical actuators. Microengineered cellular systems with a more mature contractile function have been developed in the last 5 years to overcome this limitation of stem cell–derived cardiomyocytes, while simultaneously measuring contractile endpoints with integrated force sensors/actuators and image-based techniques. Known effects of engineered microenvironments on the maturity of cardiomyocyte contractility have also been discovered in the development of these systems. Based on these discoveries, we review here design criteria of microengineered platforms of cardiomyocytes derived from pluripotent stem cells for measuring contractility with higher physiological relevance. These criteria involve the use of electromechanical, chemical and morphological cues, co-culture of different cell types, and three-dimensional cellular microenvironments. We further discuss the use and the current challenges for developing and improving these novel technologies for predicting clinical effects of drugs based on contractility measurements with cardiomyocytes differentiated from induced pluripotent stem cells. Future research should establish contexts of use in drug development for novel contractility assays with stem cell–derived cardiomyocytes.

## Introduction

Contractility of cardiomyocytes differentiated from human-induced pluripotent stem cells (hiPSCs) is attracting the attention of the drug development field as an *in vitro* approach to predict cardiac side effects of drugs (Takasuna et al., 2017; Yang and Papoian, 2018). For this use, the optimal system for measuring cellular contractility should reflect clinical drug-induced effects that are observed in patients and present a set of physiological mechanistic properties of the *in vivo* contractility of a human myocardium. In addition, practicality of experiments requires that the cellular material must stably attach to force sensors or actuators to assay contractility comprehensively because contractility measurements are mechanical endpoints of cell function with units of force (Knowlen et al., 1987). For assaying cardiac contractility, hiPSC-cardiomyocytes have the intrinsic advantage over many other cellular models of having a human genome and thereby avoid potential species-dependent differences in contractile drug responses that exist in most used models (Milani-Nejad and Janssen, 2014; Camacho et al., 2016). Furthermore, by being a live and cultured cellular system, hiPSC-cardiomyocytes offer advantages in terms of ease of handling and the avoidance of animal or human tissue usage to harvest test material. However, their high potential for contractile assays has various challenges regarding their non-physiological and immature properties, that have been identified while evaluating their use (Yang et al., 2014), and technical challenges to measure contractile functional endpoints. This article will address solutions to overcome some of these challenges in the context of platforms to assay contractility, with a view of their use to be a suitable cell-based platform for the detection of drug-induced inotropic effects (see the preceding article from the same authors). The use of hiPSC-cardiomyocytes also has limitations and challenges in assaying other cardiac properties in a physiologically relevant manner, such as metabolism, mitochondrial function, and electrophysiology. These limitations and potential strategies to solve them are reviewed in detail elsewhere (Keung et al., 2014; Li et al., 2016; White et al., 2016). However, given the potential roles of electrophysiological or metabolic effects on the pathophysiology of drug cardiotoxicity mechanisms and their effects on contractility (Barth and Tomaselli, 2009; Kolwicz et al., 2013), brief considerations on these aspects of cellular function are provided ahead. In general, the use of *in vitro* cellular systems aims to answer questions about specific mechanisms of drug effects.

## From Cells To Microengineered Devices

As detailed in part 1, platforms for assaying contractility *in vitro* with physiological relevance should provide contractile parameters that reflect cardiac function, such as force, tension, kinetics of contraction and relaxation, contraction times, synchronicity of movement, or other parameters that relate to these. The ability to perform these measurements should motivate the development and the use of cardiac platforms for contractility measurements with hiPSC-cardiomyocytes. Different platforms with these cells have been developed to measure different parameters that characterize contractility or its kinetics. **Table 1** presents different parameters that can evaluate how cellular platforms reflect a physiologically relevant function. Different platforms with hiPSC-cardiomyocytes can match contractile physiological responses and perform measurements to comprehensively evaluate the physiology of contractility (i.e., passive tension, force-load relation, force-frequency relation, force sensitivity to calcium, etc.). Overall, platforms with hiPSC-cardiomyocytes have been developed to measure physiologically relevant contractile function, and their use has high potential in drug development by overcoming the limitations of primary cells.

**Table 1:** Set of parameters obtained from *in vitro* contractility assays. We present values of these parameters for primary cells or tissue in parallel with measurements from platforms where hiPSC-cardiomyocytes were cultured in a physiologically relevant microenvironment.

		Primary cells or tissue	Achieved with iPSC-cardiomyocytes
Contractility		**Range**	**Range**
	Twitch force	44 ± 11.7 mN/mm^2^ (Hasenfuss et al., 1991)	1.3–3.3 mN/mm^2^ (Ruan et al., 2016; Sasaki et al., 2018)
	Passive tension	12.2 ± 0.86 mN/mm^2^ (Granzier and Irving, 1995)	0.4–21.5 mN/mm^2^ (Tulloch et al., 2011; Ruan et al., 2016)
	Sarcomere length	2.2 µm (relaxed length) (Bird et al., 2003)	1.65–2.1 µm (relaxed length) (Lundy et al., 2013; Ribeiro et al., 2017)
Kinetics		**Range (unloaded)**	**Range**
	Contraction velocity	106 ± 8.9 µm/s (Nishimura et al., 2004)	1–13 µm/s (Huebsch et al., 2015) (Sala et al., 2018)
	Relaxation velocity	37.5 ± 4.3 (Korte et al., 2011)	1–13 µm/s (Huebsch et al., 2015)
	Beat rate	Requires electrical pacing (Bird et al., 2003)	Can be paced (Ronaldson-Bouchard et al., 2018)
Physiology		**Profile**	**Profile**
	Force-load relation	Positive (Helmes et al., 2016)	Positive (Huebsch et al., 2016; Ronaldson-Bouchard et al., 2018)
	Force-frequency relation	Positive (Wiegerinck et al., 2009)	Positive (Ronaldson-Bouchard et al., 2018)
	Force sensitivity to calcium	nM–mM range (Bers and Perez-Reyes, 1999)	nM range (Ronaldson-Bouchard et al., 2018)
Inotropes		**Acute response**	**Acute response**
	Isoproterenol	Positive (Butler et al., 2015)	Positive (Mannhardt et al., 2016)
	Nifedipine	Negative (Nguyen et al., 2017)	Negative (Ronaldson-Bouchard et al., 2018)
	Verapamil	Negative (Nguyen et al., 2017)	Negative (Mannhardt et al., 2016)
	Thapsigargin	Negative (Kocic et al., 1998)	Negative (Ronaldson-Bouchard et al., 2018)
	Ouabain	Positive (Xiong et al., 2012)	Positive (Mannhardt et al., 2016)

**Figure 1:**
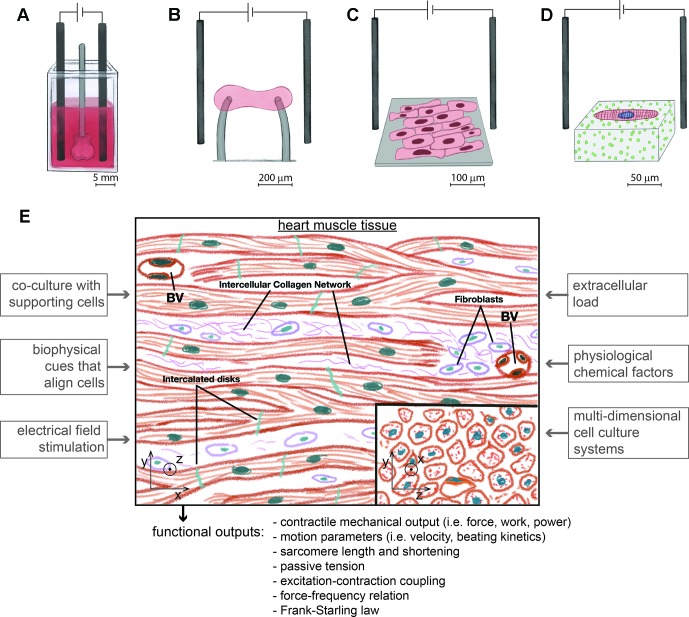
Different types of experimental platforms to assay the contractility of hiPSC-cardiomyocytes with physiological relevance, at different length scales and under electrical pacing. **(A)**. Engineered human ventricular cardiac organoid chambers contain co-cultures of hiPSC-cardiomyocytes with dermal fibroblasts organized in 3D, have the ability to be electrically paced, and produce a cardiac mechanical output, while being exposed to different levels of pressure (Li et al., 2018) **(B)**. Engineered heart tissues are organized in 3D with aligned morphologies immobilized between force sensors and also contain co-cultures of hiPSC-cardiomyocytes with fibroblasts (Hinson et al., 2015; Ronaldson-Bouchard et al., 2018). **(C)**. Monolayers of cells with an aligned rectangular morphology collectively beat and contract along the direction of alignment (Carson et al., 2016). **(D)**. Single hiPSC-cardiomyocytes are micropatterned on gels to assume a rectangular physiological shape and aligned sarcomeres. Cell contractility is measured with traction force microscopy (Ribeiro et al., 2017). **(E)**. Experimental approaches to develop platforms with hiPSC-cardiomyocytes to model cardiac contractility. The microenvironment of heart tissue is multicellular and aligned in 3D. Fibroblasts coexist with interconnected cardiomyocytes. Blood vessels (BV) are densely distributed. Inspired in the biological properties of heart tissue, different factors of an *in vitro* microenvironment enhance the maturity of hiPSC-cardiomyocytes in platforms for assaying cardiac contractility. These platforms should measure different types of contractile functional outputs. Image based on (Shepherd and Vanhoutte, 1979).

With particular interest to drug testing, developers of microengineered platforms that assay cardiac contractility with hiPSC-cardiomyocytes have done preliminary tests on their response to cardiac-specific drugs as a function of culturing cells in physiologically relevant conditions (Mannhardt et al., 2017; Li et al., 2018; Ronaldson-Bouchard et al., 2018). Ronaldson-Bouchard and colleagues have evaluated the response of 3D engineered tissues to drugs after being submitted to different types of electromechanical conditioning (Ronaldson-Bouchard et al., 2018). Conditioning cells in such tissues and doing it early in differentiation induced different responses to nifedipine, verapamil, caffeine, thapsigargin, and isoproterenol. Without the proposed conditioning, the response to any of these drugs did not correspond to clinical effects. This result demonstrated the advantage of assaying contractility with physiologically relevant cellular systems for predicting clinical effects of drugs. In a different study, Mannhardt and colleagues used the human atrial trabeculae as an experimental reference standard for evaluating the response of engineered tissues with hiPSC-cardiomyocytes to inotropic drugs (Mannhardt et al., 2017). The contractile effects of milrinone, rolipram, citalopram, nifedipine, lidocaine, formoterol, tacrolimus, digoxin, acetylsalicylic acid, and ryanodine were compared between these two platforms to evaluate how *in vitro* physiological relevance could recapitulate the response of a standard tissue. In addition to comparing drug response, the robustness and reliability of results were also evaluated, where engineered heart tissues had a better performance. In an attempt to evaluate the response of engineered tissues with clinical relevance, Li and colleagues have measured drug-induced variations of contractile parameters that can translate to *in vivo* measurements, such as pressure, stroke volume, ejection fraction, and cardiac output (Li et al., 2018). For this purpose, they exposed tissues to varied concentrations of isoproterenol, digoxin, verapamil, nifedipine, and disopyramide. These studies demonstrate the high potential of microengineered devices to measure contractile responses of hiPSC-cardiomyocytes with physiological relevance and set the tone of the future work that can validate such systems for use in drug development. Certain properties of physiological relevance can be engineered and conditioned in single cells, 2D or 3D (Schroer et al., 2018), and we review here work that has been done to develop physiologically relevant contractility assays with hiPSC-cardiomyocytes (**Figure 1**). However, many challenges still exist in the field to model the physiology of cardiac contractility, mainly when comparing the functional endpoints of hiPSC-cardiomyocytes with what can be measured with primary cells or tissues. In addition, other biological properties that affect contractility are not matured in hiPSC-cardiomyocytes, such as metabolism, bioenergetics, electrophysiology, arrhythmogenicity, and structural organization. We briefly cover these properties with reference to literature that further elaborates on them. In summary, hiPSC-cardiomyocytes enable novel methodological approaches for assaying potential drug-induced variations in cardiac contractility. Such technologies are not applicable to mature primary cardiomyocytes or isolated cardiac tissues and present an opportunity to improve the prediction of clinical drug effects. However, the use of hiPSC-cardiomyocytes presents several difficulties that arise from their immature fetal-like properties that may not emulate functional settings of human physiology.

### hiPSC-Cardiomyocytes Can Overcome Some of the Limitations of Using Primary Cellular Material

Overall, hiPSC-cardiomyocytes have different biological properties and are cultured differently from primary cardiomyocytes (Bird et al., 2003; Louch et al., 2011; Robertson et al., 2013; Yang et al., 2014; Atmanli and Domian, 2017). Specifically, the use of primary cardiomyocytes as *in vitro* contractility platforms for predicting clinical drug effects has been limited by the following factors:

- Isolation of healthy human primary cardiomyocytes is restricted to a low number of donors with limited genetic variety (Voigt et al., 2015);
- Cardiomyocytes isolated from animals lack human-specific properties (Ahuja et al., 2007);
- Cells do not last more than a few days in culture, are post-mitotic, and do not recover from standard cell freezing protocols (Louch et al., 2011);
- Expression, activity, density, and localization of T-tubules, ion channels, and of the sarcomere-based contractile machinery decrease within hours after isolation under physiological temperature (37°C), physiological extracellular calcium concentration, and without compounds that inhibit the contractile machinery (Louch et al., 2011);
- Their adhesiveness to common cell culture materials is low, specifically to force sensors or actuators, and they do not bind to other cultured cells (Bird et al., 2003);
- Their isolation requires skilled technical expertise (Louch et al., 2011).

Cardiomyocytes differentiated from hiPSCs are also post-mitotic, do not present the remaining disadvantages, but also have limitations related to their fetal-like properties (Robertson et al., 2013; Yang et al., 2014; Mannhardt et al., 2017) and functional variability (Kane et al., 2015; Huo et al., 2017). Fetal-like properties limit the extent of clinical predictivity of drug effects from experimental data, and attention must be given to the context of use in drug evaluation with these cells to ensure physiological relevance of results. However, the ability to culture easily hiPSC-cardiomyocytes for extended periods and to attach them to extracellular materials are the advantages that primarily enable their potential. Several approaches noted here for originating physiologically relevant contractile endpoints focus on enhancing the functional maturity of hiPSC-cardiomyocytes by replicating physiological microenvironments *in vitro*. Even with the increased attention of the field to the subject of maturity of hiPSC-cardiomyocytes, there are currently no well accepted thresholds of functional endpoints for defining maturation in these cells (Veerman et al., 2015; Weinberger et al., 2017; Mills and Hudson, 2019). In addition to fetal-like properties of differentiated cells, variability within lines of reprogrammed hiPSCs is a limitation for applications involving large-scale and multi-line comparisons (Tapia and Scholer, 2016; Ortmann and Vallier, 2017). However, despite these disadvantages and the complexity associated to maintaining pluripotency (Orkin and Hochedlinger, 2011), studying hiPSCs and their differentiation protocols presents an opportunity to unravel molecular mechanisms of human development and lineage specificity (Wu and Izpisua Belmonte, 2016).

Differences in cell culture between primary and hiPSC-derived cardiomyocytes also limit their characterization with techniques that have traditionally analyzed primary cardiomyocytes or cardiac tissues (Lee and Shideman, 1959; Langer, 1968; Ter Keurs et al., 1980; Bova et al., 1997; Ren and Wold, 2001; Liao et al., 2012). For example, detecting sarcomere shortening (Butler et al., 2015) and cell length variations (Bazan et al., 2009) in hiPSC-cardiomyocytes is challenging because cell edges and striated sarcomeres are not clearly detectable with optical microscopy as they are in primary cardiomyocytes (Kijlstra et al., 2015). However, these differences in determining contractility endpoints between primary cells and hiPSC-cardiomyocytes have opened new opportunities to apply microengineering and microfabrication techniques to assay the contractility of hiPSC-cardiomyocytes in a more physiologically relevant manner. Given the novelty of this field, recent advances in the development of microengineered platforms hold great promise on what can be achieved in their use in the years to come. **Table 1** shows a list of contractility parameters that can be measured with such platforms and also presents parameters measured from primary cellular materials.

Overall, the reprogramming of hiPSCs (Yoshida and Yamanaka, 2017), their differentiation toward cardiomyocytes (Yoshida and Yamanaka, 2017), and using these cells in contractility assays (Schroer et al., 2018; Yang and Papoian, 2018) are recent fields that still need improvement in differentiation protocols, genetic editing, and microengineering to enhance the physiological relevance of assays (Yang et al., 2014). In opposition, the isolation and the contractile analysis of primary cellular material from healthy hearts represent a well-established and robust experimental field with well-defined advantages and gaps. However, despite their disadvantages in not replicating crucial properties that define cardiomyocyte physiology, hiPSC-cardiomyocytes have the potential to produce results with higher reproducibility and robustness then primary tissues (Mannhardt et al., 2017). The most recently optimized platforms to assay cardiac contractility with hiPSC-cardiomyocytes resulted from combined multidisciplinary approaches in stem cell biology, genetics, bioengineering, and metrology (Godier-Furnemont et al., 2015; Ronaldson-Bouchard et al., 2018). Next, we introduce how the cell microenvironment of *in vitro* platforms can improve the physiological relevance of contractility assays with hiPSC-cardiomyocytes. We further provide practical examples of how the microenvironment must present specific characteristics to also measure force and apply load to cells within distinct systems with diverse length scales and dimensionality: single cells and cells on surfaces in two dimensions (2D) and organized in three dimensions (3D) as microtissues. This information will support developers of new methods on improving the current state of the art of contractility platforms using hiPSC-cardiomyocytes.

### Changing the Cellular Microenvironment to Assay Contractility With hiPSC-Cardiomyocytes

In general, the electromechanical microenvironment of hiPSC-cardiomyocytes can be engineered to enable cell contractile analysis (Del Alamo et al., 2016), enhance the physiological properties of their contractile machinery (Atmanli and Domian, 2017; Wanjare and Huang, 2017), and thereby overcome some of the technical limitations that arise from the biological differences between hiPSC-cardiomyocytes and primary cardiomyocytes. Specific modifications in the cellular microenvironment that enable microscopy-based mechanical analysis have also allowed measuring contractile properties with a detail that is difficult to measure with primary cells (Feaster et al., 2015; Hinson et al., 2015; Ribeiro et al., 2017; Ronaldson-Bouchard et al., 2018) and applying external loads to cells (Mannhardt et al., 2016; Lemoine et al., 2017; Ronaldson-Bouchard et al., 2018). Engineering the microenvironment of hiPSC-cardiomyocytes has been done with single cells (Hinson et al., 2015; Ribeiro et al., 2017), cell monolayers (Rao et al., 2013; Jung et al., 2016), and 3-dimensional (3D) engineered heart tissues (Nunes et al., 2013; Mannhardt et al., 2016; Ronaldson-Bouchard et al., 2018). In addition, the electromechanical microenvironment can be engineered with modifications of cell culture systems with different dimensions that require different cell numbers (Carson et al., 2016; Huebsch et al., 2016; Tiburcy et al., 2017; Ronaldson-Bouchard et al., 2018).

In addition to the electromechanical microenvironment, chemical and biological cellular microenvironments can also drive hiPSC-cardiomyocytes toward a more mature contractile performance (Correia et al., 2017; Parikh et al., 2017). Chemical microenvironments are set through the composition of the cell culture medium with more physiological carbon sources or hormones. More complex biological microenvironments involve co-culturing hiPSC-cardiomyocytes with non-myocyte cells of cardiac relevance that provide structural and paracrine support to the contractile function of cellular systems. Overall, the trend on how new platforms are being developed for contractility analysis with hiPSC-cardiomyocytes suggest that the microenvironment is a key component to design and engineer cellular systems (Ronaldson-Bouchard et al., 2018). The goal of culturing cells under specific microenvironment conditions is therefore to induce cellular physiological properties in hiPSC-cardiomyocytes that are absent when these cells are cultured in standard culture plates. Physiological relevance is of specific importance in drug development while testing for well-defined mechanistic pathways affected by the mechanisms of action of compounds. However, transferring microfabricated cellular platforms between laboratories and making them available to a wider range of users, while performing as published, is challenging because of their inherent complexity and need for specialized users (Pamies et al., 2018). This limitation may lead to a controversial predisposition of the field to use contractility assays with hiPSC-cardiomyocytes, which combine different cell types in atypical conditions, while involving specialized multidisciplinary teams for ensuring proper use and quality of devices (Li et al., 2018; Ronaldson-Bouchard et al., 2018).

### Physiologically Relevant Cellular Properties Define the use of hiPSC-Cardiomyocytes

For predicting clinically relevant contractile drug effects with hiPSC-cardiomyocyte-based platforms, cells must express the target molecules and pathways of drug candidates, along with physiological levels of expression and organization of contractile proteins. For example, β-adrenergic receptor signaling is poorly developed in early differentiated hiPSC-cardiomyocytes, which is required to model cardiotoxicity induced by β-adrenergic receptor stimulation (Jung et al., 2016). Preliminary studies show that prolonged cell culture and cellular alignment enhance the expression of β-adrenergic receptors in hiPSC-cardiomyocytes (Jung et al., 2016), thus demonstrating the potential of engineering microenvironments to increase the physiological relevance of cellular assays. In addition to other microenvironment conditions that may enhance cell physiology, other approaches involving gene-editing techniques may also contribute in ensuring the expression of target molecular pathways of drug candidates (Bassett, 2017; Li et al., 2017; Goversen et al., 2018). With gene-editing approaches, hiPSC-cardiomyocytes can ultimately elucidate on the contributions of different pathways and provide a mechanistic understanding of drug effects. To take advantage of this potential, novel isogenic hiPSC lines have been developed to enable genetic modifications, as already demonstrated in the expression of structural fluorescent markers (Drubin and Hyman, 2017; Roberts et al., 2017) that can also facilitate the analysis of contractility (Mandegar et al., 2016; Judge et al., 2017).

Different studies have also shown that hiPSC-cardiomyocytes can easily attach to a variety of extracellular materials, including Matrigel, fibronectin, laminin, vitronectin, and other extracellular components (Lundy et al., 2013; Burridge et al., 2014; Holt-Casper et al., 2015; Badenes et al., 2016; Ronaldson-Bouchard et al., 2018). This versatility in attaching to different extracellular components also enables the potential of culturing cells in a microenvironment that may recreate a cardiac extracellular matrix (Rienks et al., 2014; Wang et al., 2016; Li et al., 2018). All of these technical advantages are ideal for developing more informative *in vitro* platforms since these cells can be maintained in culture for months, while presenting cardiac-specific properties (contractility, electrophysiology, calcium signaling, etc.) that do not vary within hours or days. For example, long-term culture and stable biological properties allow measuring chronic and time-dependent contractile drug effects, and binding cells to extracellular materials enables the ability of sensing cell-generated forces (Polacheck and Chen, 2016; Ribeiro et al., 2016). However, fetal-like properties of hiPSC-cardiomyocytes are a major limitation that questions the physiological relevance of their use, and contexts of use in drug development must be defined for specific mechanisms that regulate contractility.

Novel complex and advanced platforms with hiPSC-cardiomyocytes have substantially improved on the physiological properties of these cells (Li et al., 2018; Ronaldson-Bouchard et al., 2018), but contexts of use of these systems still need to be investigated. Overall, platforms with an engineered microenvironment for analyzing cardiac contractility (**Figure 1**) can provide novel technical advantages to use hiPSC-cardiomyocytes in culture for months (Zhao et al., 2019). Ideally, with enhanced maturity that better models mechanistic physiological features, contractility can be measured to predict clinical drug effects (Kane and Terracciano, 2017; Tiburcy et al., 2017). The simplicity of protocols to thaw and culture hiPSC-cardiomyocytes can easily widen their use by different stakeholders involved in drug development that aim to assay cardiac contractility. With the current technical advances in this field, it is possible to analyze long-term and sub-chronic effects of drugs in more physiological settings that may have the potential predict clinical drug effects. Without focusing on a specific platform or cell culture system, we discuss here key design and engineering criteria of cell culture systems that have been proven to improve cell maturity to enable the ability to assay contractility with hiPSC-cardiomyocytes. Criteria consist of setting a physiological microenvironment, integrating methods to measure cell-generated forces, and ensuring good quality of the cellular material for contractility assays. These criteria can be considered when designing novel platforms for assaying the contractile changes of hiPSC-cardiomyocytes induced by drugs. We now focus on the integration of physical and electrical cues in potential platforms that can enable the evaluation of hiPSC-cardiomyocytes and on how these platforms can be used to derive contractile functional outputs.

## Increasing the Contractile Physiological Relevance of HiPSC-Cardiomyocytes by Inducing Cellular Alignment, Microfabricating 3D Constructs, co-Culturing Different Cell Types, Electrical Field Stimulation, Mechanical Load, and Physiological Chemical Factors

Overall, *in vitro* fetal-like cardiomyocytes lack physiological properties that exist in mature cardiomyocytes. Specifically for contractile function, immature fetal-like cardiomyocytes differ from mature cardiomyocytes by having a misaligned sarcomere-based contractile machinery with fetal-specific protein isoforms and shorter sarcomere length, lacking T-tubules, expressing lower levels of ion channels, not presenting a positive force-frequency relation, having poorly developed calcium signaling, different β-adrenergic signaling, and using different carbon sources (Robertson et al., 2013; Yang et al., 2014; Correia et al., 2017). Spontaneous beating, susceptibility for arrhythmogenic activity, and uncertainty on the chamber specificity that is represented in differentiated cells are practical hurdles for the clinical translation of drug evaluation studies with hiPSC-cardiomyocytes and the mechanistic interpretation of their results (Zhang et al., 2002; Ma et al., 2011; Denning et al., 2016; Kane et al., 2016). Inducing alignment of hiPSC-cardiomyocytes is the most commonly used approach in different platforms to improve the level of maturity of the contractile machinery of hiPSC-cardiomyocytes (Sheehy et al., 2014; Lundy et al., 2017), along with other microenvironmental cues that can further enhance the maturity of these cells (Lundy et al., 2017). In this section, in addition to different ways of structurally maturing cells with induced alignment, we also describe other strategies to enhance the physiological relevance of cardiac contractility assays with hiPSC-cardiomyocytes, which involve: (i) culturing cells in 3D constructs, (ii) electromechanical stimulation of cells, (iii) considering electrophysiological limitations, (iv) tuning the composition of the cell culture media, and (v) considering metabolic immaturity of cells.

### Inducing an Aligned and Rectangular Cell Morphology

Independently of chamber specificity (atrial or ventricular), an aligned rectangular and tubular cellular morphology is a basic characteristic of mature cardiomyocytes within a healthy myocardium *in vivo* or isolated from functional tissues (Louch et al., 2011; Voigt et al., 2015; Brandenburg et al., 2016). Independently of all differences between distinct approaches and platforms to enhance the maturity of hiPSC-cardiomyocytes, cell morphology, consisting of an aligned and rectangular shape, is the basic and most elementary maturation marker of hiPSC-cardiomyocytes cultured *in vitro* (Lundy et al., 2013; Nunes et al., 2013; Ribeiro et al., 2015a; Ribeiro et al., 2015b; Ruan et al., 2015; Carson et al., 2016; Huebsch et al., 2016; Jung et al., 2016; Ronaldson-Bouchard et al., 2018). Cell shape has been shown to relate *in vitro* to other cardiac functional cellular properties, such as contractility, electrophysiology, calcium signaling, beta-adrenergic signaling, excitation-contraction coupling, and the Frank-Starling law (Kijlstra et al., 2015; Ribeiro et al., 2015a; Ribeiro et al., 2015b; Huebsch et al., 2016; Jung et al., 2016). These properties reflect more mature contractile outputs when alignment of hiPSC-cardiomyocytes is promoted with extracellular physical cues. In addition, aligning these cells can decrease their susceptibility for arrhythmogenicity due to improved intercellular coupling (Wang et al., 2013), which may derive from a more matured organization of gap junctions in relation to the cell rectangular and aligned shape (Hsiao et al., 2013; Hansen et al., 2018). The relation between induced alignment/rectangular shape and enhanced maturity of other functional outputs has also been observed previously with neonatal and fetal cardiomyocytes cultured *in vitro* (Hirschy et al., 2006; Nunes et al., 2013; Ribeiro et al., 2015a; Ribeiro et al., 2015b; Ruan et al., 2015; Carson et al., 2016; Huebsch et al., 2016; Jung et al., 2016). However, cellular alignment alone, as a microenvironment cue, has been shown not to guarantee enhancement of the maturity of hiPSC-cardiomyocytes, measured through the analysis of calcium flow and expression of markers of cardiac maturity (Han et al., 2016). This study suggests that enhancement of cardiac maturity of hiPSC-cardiomyocytes may result from a combination of microenvironment properties, that also include 3D settings, co-culturing different cell types, electrical stimulation, mechanical load, and chemical factors.

An improved organization of sarcomeres aligned in series along intracellular myofibrils can be the direct outcome of engineering the microenvironment of hiPSC-cardiomyocytes in culture for cells to assume a rectangular and tubular shape. However, the mechanisms that link an improved structural organization of myofibrils to maturation require elucidation to clarify how the shape and structure of primary cardiomyocytes relate to each other *in vivo*. Recent work with hiPSC-cardiomyocytes in physiological *in vivo* microenvironment settings sheds light on how maturation relates to cell shape, structure, and function (Cho et al., 2017). The observed relationships between the formation of aligned myofibrils and the shape of cardiomyocytes in culture suggest that the interplay between extracellular factors and the contractile function of sarcomeres plays a key role in cellular alignment, shape, and myofibril organization (Engler et al., 2008; Chopra et al., 2011; Chopra et al., 2012; Feinberg et al., 2012; Kuo et al., 2012; Majkut et al., 2013; Majkut et al., 2014). In addition, mathematical models strongly support the roles of tuned extracellular rigidity, contractile function, and cell adhesions in the cytoarchitectural changes that drive an aligned rectangular shape and matured myofibril organization of cardiomyocytes (Dasbiswas et al., 2015; Lemke and Schnorrer, 2017).

Given this relationship between structural organization and a more matured contractile function, sarcomere organization can be quantified as a metric of maturation, and several structural phenotypes can evaluate cell maturity from hiPSC-cardiomyocytes with labeled contractile proteins (Pasqualini et al., 2015). These phenotypes can quantify in an unbiased manner the level of myofibril alignment, the amount of lateral registry between neighboring sarcomeres, the amount of cellular area/volume populated by sarcomeres, and the homogeneity of structural organization within the cells. Following this approach, novel computational tools based on machine learning algorithms may have a high potential to screen for more mature cellular systems (Rajaram et al., 2012; Kraus et al., 2016) as strategies for quality control (Sheehy et al., 2014; Pasqualini et al., 2015), prior to contractility assays. Quality control of cellular systems is particularly crucial with hiPSC-differentiated cells because of the high levels of variability that are often reported to exist between cell batches differentiated by different laboratories and between different cell lines (Grimm et al., 2015; Bargaje et al., 2017; Carcamo-Orive et al., 2017; Kallur et al., 2017). In general, alignment of cells to be submitted to contractile analysis can be induced with microcontact printing (Kijlstra et al., 2015; Ribeiro et al., 2015a; Ribeiro et al., 2015b), anisotropic topography of cell culture surfaces (Carson et al., 2016; Jung et al., 2016), tuned substrate rigidity (Ribeiro et al., 2015a), stretching (Chun et al., 2015), and microfabricated aligned microtissues (Huebsch et al., 2016). In addition, long-term culture of hiPSC-cardiomyocytes has been also reported to improve their structure as cells mature (Lundy et al., 2013), suggesting that inducing cellular alignment and rectangular shape *in vitro* may accelerate the maturation pathways involved in this process (Jung et al., 2016). In conclusion, these principles to induce cellular alignment should be considered in the fabrication of 3D- (**Figures 1A, B**), 2D- (**Figure 1C**), or single cell–based (**Figure 1D**) platforms to measure the contractile function of hiPSC-cardiomyocytes in more mature settings.

### Three-Dimensional Cultures With Other Supporting Cell Types and Electromechanical Stimulation

The native microenvironment of the myocardium is organized in 3D (Opie, 2004b), and *in vitro* 3D aligned constructs containing hiPSC-cardiomyocytes are among the most reliable platforms for enhancing the maturity of hiPSC-cardiomyocytes (Mannhardt et al., 2016; Li et al., 2018; Ronaldson-Bouchard et al., 2018). These constructs can be termed in various ways as ventricular cardiac organoid chambers (Li et al., 2018) (**Figure 1A**) or engineered heart tissues (Fink et al., 2000; Zimmermann et al., 2000; Hansen et al., 2010; Eder et al., 2016; Mannhardt et al., 2016) (**Figure 1B**), engineered human myocardium (Tiburcy et al., 2017), or simply cardiac microphysiological systems (Mathur et al., 2015; Lind et al., 2017). Ventricular- or atrial-like tissue constructs can be developed with different differentiation protocols and microenvironment electromechanical cues (Zhao et al., 2019). In addition, following known cellular compositions of cardiac tissue (**Figure 1E**), adding non-myocyte cells to 3D constructs, improves the physiological relevance of their contractility (Kurokawa and George, 2016; Li et al., 2018; Ronaldson-Bouchard et al., 2018).

The type of non-myocyte cells (i.e., fibroblasts, endothelial cells, stromal cells, etc.) to co-culture in 3D systems is still not consensual, and there are many promising options to consider based on published results. Fibroblasts are among the most used cell types for these co-culture applications (Zuppinger, 2016), with unclear benefits (Kurokawa and George, 2016), but non-myocyte cells originated as a byproduct of cardiac differentiations also provide improved structural support to hiPSC-cardiomyocytes in 3D and mature their function (Huebsch et al., 2016). Most importantly, co-culturing hiPSC-cardiomyocytes with non-myocyte cells present the risk of negatively affecting intercellular electrical coupling if the number of non-excitable cells exceeds critical values that lead to the formation of fibrotic tissue (van Spreeuwel et al., 2017). Besides non-myocyte cells being non-excitable, adhesions between cardiomyocytes differ from adhesions between cardiomyocytes and non-myocyte cells (Pedrotty et al., 2008). Adhesions of cardiomyocytes to non-myocyte cells affect intercellular electrical coupling. Managing the risk of decreased cardiac performance due to an excessive concentration of non-myocyte cells relies on controlling their proliferation (Pellman et al., 2016). The major role of non-myocyte cells in 3D constructs has been shown to provide structural support, thus ensuring tissue mechanical integrity (Kensah et al., 2013). 3D co-cultures of hiPSC-cardiomyocytes are usually done in collagen-based hydrogels that induce extracellular remodeling and reorganization into functional tissues (Hirt et al., 2014). Fibrinogen-based hydrogels are used to ensure tissue integrity in 3D constructs containing only hiPSC-cardiomyocytes, without the use of support cells (Hirt et al., 2014). Under these conditions, function of 3D tissues with monocultures of hiPSC-cardiomyocytes can last for several weeks without significant variations (Mannhardt et al., 2016). Other components are also used for enabling extracellular support of 3D tissues in addition to collagen- or fibrinogen-based matrices (Hirt et al., 2014). For example, a recent study with 3D tested the combinations of collagen with fibrinogen to optimize cardiac function (Kaiser et al., 2019).

In addition to support cells and extracellular matrix proteins, other extracellular cues of physiological relevance, such as mechanical load (Ruan et al., 2015; Abilez et al., 2018) and electrical stimulation (Nunes et al., 2013) also enhance the maturity of hiPSC-cardiomyocytes when delivered to 3D constructs for days (Zhao et al., 2019). For this purpose, protocols have been published to inform potential system developers on culturing hiPSC-cardiomyocytes beating against a mechanical load of controllable magnitude (Tulloch et al., 2011; Hirt et al., 2012) and in the presence of well-defined electrical stimulation (Sun and Nunes, 2017; Ronaldson-Bouchard et al., 2018; Zhao et al., 2019). A study with engineered heart tissues showed the functional effects of increasing afterload, which resembled the pathophysiology of hypertrophic cardiomyopathy, and further demonstrated the possibility to replicate physiological settings by tuning the tissue resistance to beating (Hirt et al., 2012).

Some of the most common methods for fabricating 3D cellular constructs involve soft lithography, 3D printing, laser cutting, and micromolding (Guven et al., 2015). The need for high quantities of cells is the main disadvantage of 3D constructs, which may impose a high cost to these systems if purchasing commercially available hiPSC-cardiomyocytes. Attempts to reduce cell number in 3D constructs, while maintaining a physiological function with aligned hiPSC-cardiomyocytes, have been successful (Huebsch et al., 2016), but smaller structures are more difficult to handle and functionally analyze. The field of spheroids and organoids illustrates well the difficulties to obtain contractile outputs from microcellular systems. Cardiac spheroids are promising platforms to replicate physiological microenvironments with reduced cell numbers (Giacomelli et al., 2017; Polonchuk et al., 2017; Sirenko et al., 2017; Hoang et al., 2018), but measuring contractile forces within these structures is challenging. Overall, to elucidate on the use of different systems with hiPSC-cardiomyocytes, the field requires an evaluation on their contexts of use and setting of quality control parameters to determine the benefits from the additional cost and time associated with fabricating and operating more complex approaches.

### Consideration on Electrophysiological Fetal-Like Properties of hiPSC-Cardiomyocytes

Contractility is linked to electrophysiology *via* mechanisms of excitation-contraction coupling (Eisner et al., 2017). It is thus important to consider that, unlike in the mature human heart, excitatory anatomical pace-making nodes do not impart beating in cultures of hiPSC-cardiomyocytes. In opposition to isolated primary cardiomyocytes, where spontaneous contractions indicate loss of membrane integrity or damage, hiPSC-cardiomyocytes have immature spontaneous diastolic depolarization, even when cultured in microenvironments of engineered tissues (Lemoine et al., 2018; Ulmer et al., 2018). Overall, the expression of ion currents in hiPSC-cardiomyocytes differs from ventricular cardiomyocytes (Blazeski et al., 2012) (**Table 2**), which can be improved by prolonging cells in culture or by recreating physiological microenvironments (Kim et al., 2010; Ribeiro et al., 2015b; Herron et al., 2016; Yoshida et al., 2018). However, improvements in cell maturity do not result in cells that fully recapitulate the functional properties of primary cardiomyocytes. In addition to enhanced maturation, controlling spontaneous contractions has been shown to improve the physiological relevance of the effects of inotropes in hiPSC-cardiomyocytes (Mannhardt et al., 2016).

**Table 2:** Experimentally obtained properties related to specific levels of cardiomyocyte function that can affect contractility or cellular effects of compounds. The values of properties have been published and are presented for primary cardiomyocytes, hiPSC-cardiomyocytes in 2D, and engineered heart tissues. I generally represents the current density of different regulators of electrophysiological function: sodium-calcium exchange current (I_NCX_), sodium current (I_Na_), rapidly activating component of the rectifier potassium current (I_Kr_), L-type calcium current (I_Ca,L_), inward rectifier potassium current (I_K1_), “funny” current (I_f_), slowly activating component of the delayed rectifier potassium current (I_KS_), calcium-insensitive transient outward current (I_to1_), and T-type calcium current (I_Ca,T_). MHC represents myosin-heavy chain, which affects the physiological relevance of contractility.

Functional property	Primary cardiomyocytes	hiPSC-cardiomyocytes in 2D	Engineered heart tissues
*Membrane capacitance (pF)*	∼ 200pF (Feric and Radisic, 2016)	∼ 10–55 (Feric and Radisic, 2016; Vaidyanathan et al., 2016)	∼ 28.2–47 isolated cells (Horvath et al., 2018)
*Maximal upstroke velocity (V/s)*	∼ 230–253 (Lemoine et al., 2017)	∼ 13.1–146.5 (Lemoine et al., 2017)	∼ 219 (Lemoine et al., 2017)
*Action potential duration (ms)*	∼ 228–411(Feric and Radisic, 2016; Horvath et al., 2018)	∼ 200–500 (Feric and Radisic, 2016)	∼ 206–422 (Horvath et al., 2018)
*Action potential amplitude (mV)*	∼ 94.3–104.8 (Lemoine et al., 2017)	∼ 88.1–116 (Lemoine et al., 2017)	∼ 102.7(Lemoine et al., 2017)
*Resting membrane potential (mV)*	∼ -72.6−90 (Feric and Radisic, 2016; Lemoine et al., 2017)	∼ -37−70.5 (Feric and Radisic, 2016; Lemoine et al., 2017)	∼ -73.5(Lemoine et al., 2017)
*I* *_NCX_* *(pA/pF)*	∼ -1.0 (Koivumaki et al., 2018)	∼ -1.2−6.9 (Barbuti et al., 2016)	Expression levels of NCX-like 2D (Mannhardt et al., 2016)
*I_Na_ (pA/pF)*	∼ -20.2–−14.3 (Lemoine et al., 2017)	∼ -10.3 (Lemoine et al., 2017)	∼ -18.5(Lemoine et al., 2017)
*I* *_Kr_* *(pA/pF)*	∼ 0.25–0.6 (Casini et al., 2017)	∼ 0.18–2.5 (Casini et al., 2017)	Magnitude reported ∼ 1/3 < primary tissue (Lemoine et al., 2018)
*I* *_Ca,L_* *(pA/pF)*	∼ -3.8–10.2 (Casini et al., 2017)	∼ -6.6–58 (Casini et al., 2017)	Magnitude reported 1.5× > 2D (Uzun et al., 2016)
*I* *_K1_* *(pA/pF)*	∼ -3.6–32.1 (Casini et al., 2017)	∼ -0.8–5.1 (Casini et al., 2017)	1/2 magnitude recorded with 2D (Horvath et al., 2018)
*I* *_f_* *(pA/pF)*	∼ -1.18 (Casini et al., 2017)	∼ -0.9–4.1 (Casini et al., 2017)	Magnitude reported to be 5× > primary tissWue (Lemoine et al., 2018)
*I* *_KS_* *(pA/pF)*	∼ 0.18 (Casini et al., 2017)	∼ 0.22–2.9 (Casini et al., 2017)	Magnitude reported to match primary tissue (Lemoine et al., 2018)
*I* *_to1_* *(pA/pF)*	∼ 4.4–10.6 (Casini et al., 2017)	∼ 1.3–1.9 (Casini et al., 2017)	–
*I* *_Ca,T_* *(pA/pF)*	Expressed in immature cells and pacemaker cells (Mesirca et al., 2014)	∼ -2.1 ± 0.8 (Zhao et al., 2018)	Magnitude like 2D (Uzun et al., 2016)
*Relative expression of *α*- adrenoceptor 1A*	∼ 100 (Földes et al., 2014)	Absent (Földes et al., 2014)	–
*Relative expression of* α*- adrenoceptor 1B*	∼ 10,000 (Földes et al., 2014)	∼ 100–1,000 (Földes et al., 2014)	–
*Relative expression of β1-adrenoceptors *	∼ 5,000 (Jung et al., 2016)	Residual to ∼ 3,000 (Jung et al., 2016)	Improved β-adrenergic response relative to 2D (Weinberger et al., 2017)
*Relative expression of β2- adrenoceptors *	∼ 5,000 (Jung et al., 2016)	∼ 2,500–9,000 (Jung et al., 2016)	Improved β-adrenergic response relative to 2D (Weinberger et al., 2017)
*Relative expression of β3- adrenoceptors *	∼ 1,000 (Jung et al., 2016)	Residual-∼ 500 (Jung et al., 2016)	Improved β-adrenergic response relative to 2D (Weinberger et al., 2017)
*Cell shape and size*	Tubular, long, and narrow (length: 50–100 µm, diameter: 10–25 µm) (Opie, 2004a)	Circular (variable area: 1,000–1,800 µm^2^) (Lewandowski et al., 2018)	Tubular, long, and narrow, but not as large as primary cells (Weinberger et al., 2017)
*Organization of mitochondria *	Distributed proximally to myofibrils, occupy ∼ 20 to 40% of cell (Yang et al., 2014)	Irregular cytoplasmic distribution, less dense than in primary cells (Yang et al., 2014)	Systematically present, but with immature organization (Mannhardt et al., 2016)
*Relative expression of Connexin-43 expression*	∼ 3.2 (Lewandowski et al., 2018)	∼ 1 (Lewandowski et al., 2018)	Increased when exposed to chronic electrical stimulation (Chiu et al., 2011)
*Connexin-43 localization*	Polarized to intercalated discs (Stroemlund et al., 2015)	Intracellular localization and homogeneous distribution along the cell–cell interface (Seki et al., 2014)	No differences between end-to-end and lateral cell–cell contacts (Lemoine et al., 2017)
*Titin isoforms*	N2B > N2BA (Yang et al., 2014)	N2BA > N2B (Yang et al., 2014)	No reported data. Capable of detecting effects caused by titin mutations (Hinson et al., 2015)
*α-MHC/β-MHC*	< < 1 (Yang et al., 2014)	< 1 (Yang et al., 2014)	< Than 2D (Schaaf et al., 2011)
*T-tubules*	Highly abundant and homogeneously localized in proximity to Z-lines (Mitcheson et al., 1998)	Absent (Veerman et al., 2015)	Reported in one study (Ronaldson-Bouchard et al., 2018)

Cell-intrinsic automaticity arises from ectopic expression of the sarcolemmal “funny” current (or “If”) activated near resting potential, with an additional contribution from ionic cycling involving intracellular calcium stores (Yaniv et al., 2015). In the postnatal mouse ventricle, expression of the HCN4 ion channels, the major molecular component of If, is confined spatially to the cardiac conduction system (Liang et al., 2013), which extrinsically transmits a chamber-specific contraction sequence. However, in the prenatal mouse heart for example, expression of HCN4 is distributed more widely, and isolated ventricular myocytes are spontaneously contractile like hiPSC-cardiomyocytes (Yasui et al., 2001). This suggests that hiPSC-cardiomyocytes exhibit an “immature” functional phenotype. Indeed, while HCN4 expression in human adult ventricle is minimal, levels of HCN4 mRNA in fetal ventricle and hiPSC-cardiomyocytes are both much higher (Huo et al., 2017). A strong role of HCN4 in spontaneous beating of hiPSC-cardiomyocytes is further supported by effects of selective channel blockers: HCN4 blockers such as ZD7288 and ivabradine markedly slow spontaneous beating in hiPSC-cardiomyocytes while causing only small effects on contraction or field potential duration and do not lengthen the refractory period during electrical stimulation as observed for hERG blockers (Kitaguchi et al., 2017; Zeng et al., 2018). For example, the use of ivabradine at concentrations within the sub-µM range decreases spontaneous contractions in engineered heart tissues, yielding more physiological rate-dependent inotropic responses (Mannhardt et al., 2016). However, despite their enhanced maturity in engineered heart tissues and reduction of spontaneous contractions, electrophysiological characterization of cells in these conditions shows lower density of ion channels involved in repolarization relative to human left ventricular cells (Lemoine et al., 2018). During spontaneous beating, hERG blockers predominantly affect contraction duration (Asakura et al., 2015) and retard spontaneous beat rate primarily by increasing the period during which cells are refractory to subsequent excitation (Rast et al., 2016). In addition to the established relationship between myocardial contractile force and beat rate (Endoh, 2004), the need to mechanistically deconvolve rate from other metrics of spontaneously beating hiPSC-cardiomyocytes, such as field potential duration, has recently been highlighted (Rast et al., 2016). It is also experimentally unconfirmed whether the presence of ectopic If during a ventricular action potential would directly alter the waveform in ways that confound interpretation of downstream inotropy data, although *in silico* modeling suggests that this is indeed possible (Paci et al., 2015). It is not clear to which extent the electrophysiological immaturity of hiPSC-cardiomyocytes affects the maturity of their contractile function when cultured in more physiological microenvironments, specifically for cells with lower repolarization reserves. In general, further experimentation to pharmacologically deconvolve inotropy from chronotropy in hiPSC-cardiomyocytes is necessary, as is the refinement of techniques to stimulate action potentials independently of If (Rehnelt et al., 2017; Zeng et al., 2018). However, lack of maturity of cellular systems can affect drug responses and lead to results with no clinical translation, mainly if drug mechanisms of action or adverse effects rely on biological mechanisms that define cardiac maturity and may be absent from specific systems. Therefore, distinct parameters of cellular function (**Table 2**) should be characterized in systems with hiPSC-cardiomyocytes to increase the level of confidence in their use.

### Culture Medium to Enhance Cell Physiology and Tune Cardiomyocyte Differentiation

Changing the chemical composition of the culture medium during and after the differentiations of hiPSC-cardiomyocytes with small molecules that regulate cardiac developmental pathways and physiological carbon sources, hormones, matrix proteins, and growth factors can also enhance the maturity of these cells (Maillet et al., 2013; Burridge et al., 2014; Birket et al., 2015; Bedada et al., 2016; Cadet and Kamp, 2017; Correia et al., 2017). Overall, independently of cellular immaturity, the most commonly used differentiation protocols yield hiPSC-cardiomyocytes with ventricular-like profiles (Huo et al., 2017; Horvath et al., 2018). However, specific differentiation protocols involving chemically defined medium compositions can yield cells with different chamber-specific characteristics or maturity levels (Talkhabi et al., 2016; Pei et al., 2017; Hu et al., 2018). Functional data from cells under tuned chemical *stimuli* suggest that different contexts of use can be better represented experimentally with the right chemical microenvironment. For example, Birket and colleagues (Birket et al., 2015) optimized a combination of thyroid hormone, the glucocorticoid dexamethasone, and insulin growth factor-1 to improve several properties of mature cardiomyocytes, which was reported to enable the detection of contractile defects induced by decreased expression of myosin-binding protein C. In addition, Parikh and colleagues (Parikh et al., 2017) recently optimized a combination of hormones and matrix proteins to robustly induce the formation T-tubules. With T-tubules, hiPSC-cardiomyocytes may be more appropriate for modeling a cardiac contractile function based on a more mature excitation-contraction coupling, but these T-tubulated cells are still not able to fulfil this potential (Cadet and Kamp, 2017) and may require further optimization.

In general, chemical cues are the main players in differentiation protocols (Lian et al., 2012; Burridge et al., 2014) and should also be considered while designing the culture medium to be used during contractility assays (Schocken et al., 2017) or for preparing cells to be assayed (Pei et al., 2017). The various differentiation protocols for hiPSC-cardiomyocytes have already been reviewed in detail elsewhere (Burridge et al., 2014; Talkhabi et al., 2016; Lewandowski et al., 2017) and is not our focus here. In general, all differentiation protocols can yield hiPSC-cardiomyocytes with variable properties, and it is difficult to compare hiPSC-cardiomyocytes differentiated with distinct methods. Therefore, variations in differentiation protocols can be difficult to distinguish from differences between the genetic backgrounds of distinct cell lines (Sanchez-Freire et al., 2014; Carcamo-Orive et al., 2017). Variations between hiPSC-cardiomyocytes of different commercial sources clearly demonstrate how the sensitivity of cellular responses to drugs can differ between lines that aim to represent a healthy baseline function (Blinova et al., 2017; Huo et al., 2017). Commercial vendors differentiate cells differently from one another with proprietary media compositions but can follow quality control steps that may be impractical in research laboratories to systematically produce cells with invariable properties. In summary, experiments for assaying the effects of drugs with hiPSC-cardiomyocytes should take into consideration their differentiation protocols and origin. In addition, the medium to be used in culture or during experimental assays should emulate metabolic pathways and chemical microenvironment conditions that play roles in the cellular mechanisms of drug response (Ellen Kreipke et al., 2016; Maillet et al., 2016; Necela et al., 2017; Hu et al., 2018).

The presence of mechanistic cellular properties known to enable drug effects can define the contexts of use of *in vitro* drug development tools (Clegg and Mac Gabhann, 2015). The developers of contractility platforms with hiPSC-cardiomyocytes have demonstrated their potential for assaying drugs in physiological microenvironments (Mannhardt et al., 2017; Li et al., 2018; Ronaldson-Bouchard et al., 2018), but future work must further evaluate the standard operation procedures for cell differentiation and maintenance and set contexts of use for these systems. In addition to being functionally different from primary cells (**Table 1** and **2**), published results clearly show a high variability in functional endpoints of hiPSC-cardiomyocytes (**Table 2**), which most likely derives from differences between laboratories in differentiating hiPSCs and maintaining differentiated cells (Yassa et al., 2018; Biendarra-Tiegs et al., 2019). In general, for assessing drug cardiac safety with novel drug development tools, such as contractility assays, one must first define their contexts of use to understand to which extent they may predict clinical effects (Amur et al., 2015; Sauer and Porter, 2018). Validating contractility assays should involve a multi-stakeholder consortia with regulatory agencies, industry, and academia, as previously done for biomarker qualification (Amur et al., 2015) and more recently in the CiPA initiative (Wallis et al., 2018). Such a concerted effort would clarify on proper laboratory practices, experimental conditions, quality control criteria for cellular material, and proper testing of compounds (Pamies et al., 2018; Wallis et al., 2018).

### Consideration on the Bioenergetics and Metabolism of hiPSC-Cardiomyocytes

Mitochondria, also known as sarcosomes, in primary mature cardiomyocytes contain most of the components involved in oxidative phosphorylation in aerobic respiration, producing the high levels of energy that fuels the contractile function (Legato, 1973; Opie, 2004a; Yang et al., 2014). Mitochondria are organized in intimate contact with the contractile machinery in primary cardiomyocytes, occupying around 35% of the highly dense intracellular space (Legato, 1973). Mitochondria in mature cardiomyocytes are also found aggregated in pools near the extremities of elongated nuclei (Legato, 1973). Images of hiPSC-cardiomyocytes acquired with electron microscopy or fluorescence microscopy after labeling mitochondria show low mitochondrial density and lack of intimate proximity to myofibrils (Lundy et al., 2013; Ribeiro et al., 2015a). The disparity in mitochondrial number and organization between hiPSC-derived and mature cardiomyocytes, in addition to differences in metabolism (Ulmer and Eschenhagen, 2019), reveals the energetic immaturity of hiPSC-cardiomyocytes. Therefore, these cells may not detect cardiac drug effects that rely on metabolic pathways. Energy production in hiPSC-cardiomyocytes results from glycolysis and oxidative phosphorylation of mainly lactate (Hattori et al., 2010; Lopaschuk and Jaswal, 2010; Rana et al., 2012; Ellen Kreipke et al., 2016), while the primary source of energy in healthy mature cardiomyocytes originates from mitochondrial aerobic metabolism, being approximately 90% derived from fatty acid oxidation into acetyl-CoA prior to integration in the citrate cycle (Harris and Das, 1991; Opie, 2004a). Recent work has demonstrated the possibility to enhance the metabolic maturity of hiPSC-cardiomyocytes through induction of fatty acid metabolism (Hu et al., 2018; Nose et al., 2018; Ramachandra et al., 2018) and with 3D microenvironments that can enable increased contractile work (Correia et al., 2018; Ulmer et al., 2018). Such strategies can improve the use of hiPSC-cardiomyocytes to evaluate drug effects that mechanistically depend on metabolism.

## Engineering the Image-Based Analysis of Cardiac Function *In Vitro*

Different approaches have been developed to directly analyze the contractility of hiPSC-cardiomyocytes by measuring the output of the contractile machinery (Mannhardt et al., 2016; Ribeiro et al., 2017; Tiburcy et al., 2017; Schroer et al., 2018), excitation-contraction coupling (Kane et al., 2015), force-frequency relation (Godier-Furnémont et al., 2015), load-velocity relation (Blazeski et al., 2012), Frank-Starling effect (Huebsch et al., 2016), etc. Since contractility results in a mechanical output, most of these assays involve image-based methodologies to relate morphological variations with cell-generated forces (Mannhardt et al., 2016; Ribeiro et al., 2017; Tiburcy et al., 2017), where cells stably attach to force sensors. Ideally, systems should be designed to sense force and load relative to baseline unloaded states. However, other assays can be adapted to image-based approaches to enable higher throughput capabilities. For example, use of the patch-clamp is the state of the art method for fully characterizing the electrophysiology of cardiomyocytes (Bebarova, 2012), but imaging the variation of intensity of voltage-sensitive dyes can also provide electrophysiological information about labeled cells in higher throughput (Bedut et al., 2016). In addition, video microscopy has been a method of choice for detecting cardiomyocyte contractility *in vitro*.

Recent advances in camera performance and computer power enabled detailed kinetic analysis of the contraction and relaxation process based on movie images with high spatiotemporal resolution (Hayakawa et al., 2014). By detecting hiPSC-cardiomyocyte shortening/deformation or its rate of movement from image-based methodologies, contractile functional outputs can be evaluated in relative value, e.g., % control. Several studies have discussed the importance of evaluating contractility of hiPSC-cardiomyocytes by measuring the force developed during contractile cycles after culturing cells on elastic substrates or with deformable constructs that possess known elastic moduli (Mannhardt et al., 2016; Ribeiro et al., 2017; Tiburcy et al., 2017). If not using cytotoxic fluorescent dyes, image-based assays have the advantage of being non-destructive and minimally invasive, allowing the analysis of acute and chronic drug effects in the same cell or tissue without damaging its function (Ribeiro et al., 2017). Other advantages of imaging techniques for evaluating the contractility of hiPSC-cardiomyocytes include: 1) the simplicity of imaging cells cultured in any platform, including single cell arrays, 2D and 3D platforms; 2) no need for extensive calibration steps before measurements, while assuming that cells have homogeneous mechanical properties; and 3) the miniaturization of cell contraction measurements in micron-scale chambers, e.g., inside the narrow chamber in an organ-on-a-chip, where it is difficult to integrate more traditional force or tension sensors (Mathur et al., 2015; Mathur et al., 2016; Ronaldson-Bouchard et al., 2018). In summary, most microfabricated platforms are designed to be compatible with standard live-cell microscopy techniques, and we review some design features of devices that allow image-based characterization of cultured hiPSC-cardiomyocytes.

The most common and trivial approaches to allow imaging of cells in a device involve the use of transparent materials, such as gels, silicones, glass, polystyrene, or acrylics, while also ensuring an accessible optical path between imaged cells and the microscope objectives. However, the surface of the material of choice must be engineered to enable stable and durable cell adhesion (Polacheck and Chen, 2016; Ribeiro et al., 2016), which is required for long-term culture. Microphysiological systems are a good example of such devices (Mathur et al., 2015), where cell cultures are encapsulated in transparent microfluidic chambers and compounds, fiducial labels, or fluorescent dyes can be perfused for testing. The ability to image cells in a device with microscopy is sufficient for image-based assays that consist of analyzing intensity flow of a fluorescent signal or other imaged markers. However, comprehensive contractile assays with cellular systems require the incorporation of features for sensing active contractile force and passive tension, and for applying a mechanical load for hiPSC-cardiomyocytes to beat against (**Figure 1**). Motion tracking must present further capabilities to calculate contractility, preload, and afterload to provide a complete characterization of cardiac inotropy. Strategies for sensing force in contractile assays with hiPSC-cardiomyocytes have already implemented in different platforms that use single cells (Ribeiro et al., 2017), 2D cell layers (Artmann et al., 2016), or 3D constructs (Mannhardt et al., 2016; Li et al., 2018; Ronaldson-Bouchard et al., 2018). Sensing passive tension, which relates to preload and afterload, requires measuring the mechanical state of cellular systems when cells are diastolic/relaxed by comparing it to an unloaded baseline state (Linder et al., 2010; Godier-Furnémont et al., 2015; Mannhardt et al., 2016). In addition, mechanical load can also be applied to different dimensions of cellular organization by varying extracellular rigidity/flexibility (Ribeiro et al., 2015a), immobilizing cells/constructs between flexible posts of controllable spring constant (Mannhardt et al., 2016), or by embedding a force sensory feature within cardiomyocyte cultures/constructs (Tiburcy et al., 2017). Overall, the contractile analysis of hiPSC-cardiomyocytes requires additional microfabrication efforts on platforms for maturing these cells, and traditional cell culture plates do not enable the ability to analyze the contractile performance of hiPSC-cardiomyocytes.

## Remaining Challenges and Conclusions

All models utilized in drug development have limitations that impact their ability to translate to humans. Overall, cellular models do not replicate a complete regulatory physiology of *in vivo* models and must be evaluated to define their context of use. Many of the limitations of models with hiPSC-cardiomyocytes are common across models of other tissue systems and must be identified and assessed. Different questions need to be answered before safely and robustly using hiPSC-cardiomyocytes in the field of drug development, and further research is necessary for this purpose. What is the appropriate duration of exposure, and how many exposure multiples are required for a model? Will the model pick up effects that are acute, chronic, or both? Will the model detect direct and indirect effects (or on-target *vs*. off-target)? Does the model generate appropriate cellular metabolites? Does the model have appropriate basal/tonic levels of cellular and functional activity? Does the model show clinical relevance in drug response? Does the model express the relevant molecular pathway of the drug target? Specifically, for myocardial contractility, if a cellular model is not under load, what is the impact of an unloaded model on the contractility endpoint? Do cells self-organize to generate a constant intracellular loading state, or is it variable for construct-to-construct or cell-to-cell? It is acknowledged that no model can adequately address all concerns, but understanding the strengths and weaknesses of a specific model is paramount to understand its value to predict drug effects in humans and to be able to identify gaps in translation. An ideal model would have few gaps in its ability to translate to human and would have strategies in place to minimize the impact of its recognized limitations.

## Author Contributions

AR, BG, ME, SE, CF, LG, GG, JK, SP, JP, MB, KC, YK and BB contributed equally to this manuscript.

## Funding

The writing of this manuscript has been funded in part with federal funds from the National Cancer Institute, National Institutes of Health, under contract no. HHSN261200800001E. This project has been funded in whole or in part with federal funds from the National Cancer Institute, National Institutes of Health, under Contract No. HHSN261200800001E. FDA Office of the Chief Scientist via Challenge Grant number 848

## Disclaimer

The content of this publication does not necessarily reflect the views or policies of the Department of Health and Human Services, including the FDA, nor does mention of trade names, commercial products, or organizations imply endorsement by the U.S. Government. The content of this publication does not necessarily reflect the views or policies of the Department of Health and Human Services, nor does mention of trade names, commercial products, or organizations imply endorsement by the U.S. Government. This research was supported [in part] by the Developmental Therapeutics Program in the Division of Cancer Treatment and Diagnosis of the National Cancer Institute.

## Conflict of Interest Statement

MB was employed by Gentech. BG was employed by Pharma GmbH & Co KG. CF and GG were employed by AbbVie. LG was employed by Leidos Biomedical Research Inc. KC was employed by GlaxoSmithKline plc.

The remaining authors declare that the manuscript was written in the absence of any commercial or financial relationships that could be construed as a potential conflict of interest.
